# A Multimethod Evaluation of Tobacco Treatment Trial Recruitment Messages for Current Smokers Recently Diagnosed With Cancer: Pilot Factorial Randomized Controlled Trial

**DOI:** 10.2196/37526

**Published:** 2022-08-24

**Authors:** Jordan M Neil, Christian Senecal, Lauren Ballini, Yuchiao Chang, Brett Goshe, Efren Flores, Jamie S Ostroff, Elyse R Park

**Affiliations:** 1 Tobacco Settlement Endowment Trust Health Promotion Research Center Stephenson Cancer Center University of Oklahoma Health Sciences Center Oklahoma City, OK United States; 2 Department of Community Health Tufts University Medford, MA United States; 3 Division of General Internal Medicine Harvard Medical School/Massachusetts General Hospital Boston, MA United States; 4 Department of Psychiatry Harvard Medical School/Massachusetts General Hospital Boston, MA United States; 5 Department of Radiology Harvard Medical School/Massachusetts General Hospital Boston, MA United States; 6 Department of Psychiatry and Behavioral Sciences Memorial Sloan Kettering Cancer Center New York, NY United States

**Keywords:** teachable moment, cancer, tobacco treatment trial, smoking, message framing, recruitment

## Abstract

**Background:**

A cancer diagnosis can catalyze motivation to quit smoking. Tobacco treatment trials offer cessation resources but have low accrual rates. Digital outreach may improve accrual, but knowledge of how best to recruit smokers with recent diagnoses is limited.

**Objective:**

This study aims to identify the message frames that were most effective in promoting intent to talk to a physician about participating in a tobacco treatment trial for smokers recently diagnosed with cancer.

**Methods:**

From February to April 2019, current smokers diagnosed within the past 24 months were recruited from a national web-based panel for a multimethod pilot randomized trial (N=99). Participants were randomized to a 2×3 plus control factorial design that tested 3 unique message frames: proximal versus distal threats of smoking, costs of continued smoking versus benefits of quitting, and gains of participating versus losses of not participating in a tobacco treatment trial. The primary outcome was intent to talk to a physician about participating in a tobacco treatment trial. In phase 1, the main effect within each message factor level was examined using ANOVA and compared with the control condition. Other message evaluation and effectiveness measures were collected and explored in a multivariable model predicting intent to talk to a physician. In phase 2, open-text evaluations of the messages were analyzed using natural language processing software (Leximancer) to generate a thematic concept map and Linguistic Inquiry Word Count to identify and compare the prevalence of linguistic markers among message factors.

**Results:**

Of the 99 participants, 76 (77%) completed the intervention. Participants who received the cost of continued smoking frame were significantly more likely to intend to talk to their physician about participating in a tobacco treatment trial than those who received the benefits of the quitting frame (mean costs 5.13, SD 1.70 vs mean benefits 4.23, SD 1.86; *P*=.04). Participants who received the proximal risks of continued smoking frame were significantly more likely to seek more information about participating (mean distal 4.83, SD 1.61 vs mean proximal 5.55, SD 1.15; *P*=.04), and those who received the losses of not participating frame reported significantly improved perceptions of smoking cessation research (mean gain 3.98, SD 0.83 vs mean loss 4.38, SD 0.78; *P*=.01). Male participants (*P*=.006) and those with greater message relevancy (*P*=.001) were significantly more likely to intend to talk to their physician. Participants’ perceptions of their smoking habits, as well as their motivation to quit smoking, were prevalent themes in the open-text data. Differences in the percentages of affective words across message frames were identified.

**Conclusions:**

Multimethod approaches are needed to develop evidence-based recruitment messages for patients recently diagnosed with cancer. Future tobacco treatment trials should evaluate the effectiveness of different message frames on smoker enrollment rates.

**Trial Registration:**

Clinicaltrials.gov NCT05471284; https://clinicaltrials.gov/ct2/show/NCT05471284

## Introduction

### Background

Continued cigarette smoking is prevalent in approximately 10% to 30% of patients with recently diagnosed cancer [1,2]. Persistent smoking after diagnosis is associated with numerous adverse outcomes, including decreased treatment effectiveness, increased risk of recurrence, development of second primary cancer, and poorer overall survival outcomes [3-7]. The prevalence of smoking among patients with cancer underlines the need for timely tobacco treatment. Smokers are more likely to attempt to quit immediately after a diagnosis, suggesting that a diagnosis can serve as a *teachable moment* for smoking cessation, in which the motivation to quit is temporarily increased [8,9].

A way of leveraging this teachable moment is to enroll patients with a recent diagnosis in a tobacco treatment clinical trial. Tobacco treatment trials provide evidence-based pharmacological and behavioral therapies that personalize behavioral treatment content to address the concerns and motivations unique to smokers with cancer. However, accrual for tobacco treatment trials is suboptimal [10]. To leverage this teachable moment, as well as to attempt to overcome accrual challenges, the proactive recruitment of smokers through digital outreach offers promise. One such digital recruitment strategy is the dissemination of brief, patient-centered videos featuring clinicians describing the purpose of the trial and its relevance to the patient. This form of outreach permits investigators to deliver targeted trial information to potentially eligible smokers soon after diagnosis, when the motivation to quit may be the highest. However, to date, there has been a limited empirical examination of what content is most effective for inclusion in these outreach videos.

Although a recent diagnosis may provide an opportunity to promote cessation, it is also a time wrought by stress, guilt, stigma, and fatalism among many patients with cancer who smoke [10-16]. As such, recruitment message content promoting smoking cessation and trial participation must balance the appropriate amount of risk and benefit information to encourage participation in tobacco treatment trials.

Health communication theories can inform the content that should be used in digital outreach videos. The construal-level theory proposes that temporal distance determines how we evaluate outcomes [17]. Thus, near or more proximal outcomes are perceived more concretely, whereas distal outcomes are more abstract. Within the context of risk assessment, message cues that prompt judgments of more immediate health risks (eg, daily), compared with more long-term risks (eg, yearly), have been demonstrated to increase risk perception more effectively [18]. For individuals with a recent cancer diagnosis, it is important to understand whether smoking outcomes associated with a current diagnosis (eg, worse treatment outcomes), compared with the prospect of a future diagnosis (eg, recurrence or new primary cancer), are stronger motivators for trial enrollment and cessation initiation.

The prospect theory has been extensively studied in the context of smoking cessation [19-25]. The theory offers a framework within which to understand how individuals evaluate equivalent health messages, depending on how those messages are framed. Gain-framed messages present the likelihood of attaining desirable outcomes, whereas loss-framed messages present the likelihood of avoiding undesirable outcomes [26]. Past studies have found that gain-framed messages are more effective at conveying the short-term benefits of cessation [19]; however, there has been limited investigation into whether this strategy is as effective among patients with a recent cancer diagnosis. This is an important area of inquiry as quitting can result in important short-term benefits by reducing cancer treatment side effects, as well as improving overall energy levels and reducing levels of stress [4,8]. Determining whether to frame the benefits of cessation or the costs of not quitting on these short-term outcomes can act as an important mechanism for motivating cessation and trial enrollment during cancer treatment.

A recent investigation has explored whether it is more effective to use gain- versus loss-framed recruitment messages to motivate patient participation in a tobacco treatment clinical trial for individuals undergoing lung cancer screening (authors blinded for review). Although framing did not significantly alter motivation among smokers, it may have been more effective after a recent cancer diagnosis. The prospect theory offers contextual understanding as to why the utility of each message frame often depends on the type of health decision for which they are presented. For example, gain-framed messages are more successful at encouraging risk-averse choices, whereas loss-framed messages are more successful at motivating choices in which the outcome is more uncertain or risky [26]. However, little is known about whether these choice motivations are influenced by greater residual risk perception (ie, an active cancer diagnosis). To the best of our knowledge, no previous study has investigated gain- versus loss-framed recruitment messages within a population of patients with cancer to motivate participation in tobacco treatment trials.

### Objective

The objective of this study was to conduct a pilot factorial randomized trial to identify the message frames that are most effective in promoting participation in a tobacco treatment trial for current smokers recently diagnosed with cancer. To do so, we used a multimethod approach to evaluate 3 different message frames across evaluation, effectiveness, and outcome measures. We combine findings from a message design experiment with textual analytic software to provide a holistic understanding of how message frames may or may not differentially affect tobacco treatment trial participation within the context of a cancer diagnosis.

## Methods

### Ethics Approval

Participants received a small compensation for their participation, and institutional review board approval was obtained from Massachusetts General Brigham Hospital (#2018P002035) before data collection began.

### Sample and Procedures

From February to April 2019, a total of 99 participants were recruited from Dynata Panels, a proprietary opt-in web-based panel company, to complete a 20-minute survey. Participants were required to be English speaking, have a recent cancer diagnosis (within the past 24 months), be aged >18 years, and report any cigarette use within the past 30 days.

Participants were randomly assigned to 1 of 9 conditions as part of a 2×3 plus control factorial design. The factorial design is presented in Table 1. The first factor tested framing of the threat of continued smoking (distal vs proximal); the second factor tested framing of the response efficacy to quitting smoking (costs of continued smoking vs benefits of quitting), and the third factor tested framing of the response efficacy of participating in a cessation study (gains of participating in a smoking cessation study vs losses of not participating in a smoking cessation study). The control condition was a kernel message that included study information present in all conditions but did not include any of the message factors (described in detail in the *Stimuli* section). All participants completed premessage survey measures. After viewing 1 of the 9 videos, participants immediately completed postmessage survey measures, including open-text evaluation responses.

**Table 1:** Intervention conditions (3-factor, fully crossed factorial design).

Condition	Threat of continued smoking (distal vs proximal)	Response efficacy to quit smoking (cost vs benefit)	Response efficacy of participating in the study (gain vs loss)
1	Proximal	Cost	Loss
2	Proximal	Cost	Gain
3	Proximal	Benefit	Gain
4	Proximal	Benefit	Loss
5	Distal	Cost	Gain
6	Distal	Cost	Loss
7	Distal	Benefit	Loss
8	Distal	Benefit	Gain
9 (control)	N/A^a^	N/A	N/A

^a^N/A: not applicable.

### Stimuli

A total of 9 videos were created specifically for this study with the aim of selecting 1 video for use as part of the primary video recruitment strategy in the parent trial (SmokeFree Support Study 2.0). Each video comprised an oncologist speaking directly into the camera and was segmented into six sections, including four kernel sections that all videos possessed: (1) introducing the aims of the *Smoke Free Support Study*, (2) confirming the patient as eligible because of their recent cancer diagnosis and smoking status, (3) describing resources available in the study intervention (ie, access to remote counseling and nicotine replacement therapy), and (4) expectation setting that a study team member would contact the patient in the future to discuss willingness to participate.

Regarding the threat of continued smoking factor, the distal frame read as follows:

Every year, patients with cancer have worse outcomes because they keep smoking. By continuing to smoke, you reduce the effectiveness of your care, which means your cancer may come back and you may develop a new cancer at a later date.

The proximal frame read as follows:

Every day, patients with cancer have worse outcomes because they keep smoking. By continuing to smoke, you reduce the effectiveness of your care, which means your cancer may keep growing and you may be less likely to respond to your treatment.

For the quitting smoking factor, the costs of not quitting frame read as follows:

We want you to be aware that continuing to smoke after your cancer diagnosis can cause you to experience more side effects, increase your anxiety and stress, and have less energy.

The benefits of quitting frame read as follows:

We want you to be aware that stopping smoking after your cancer diagnosis can cause you to experience fewer side effects, decrease your anxiety and stress, and have more energy.

For the participation factor, the loss frame read:

The not-so-good news is, quitting, or even reducing the number of cigarettes you smoke each day could be more difficult without the support of our study. In fact, the Smoke Free Support Program has shown that the average patient is 3 times less likely to successfully quit smoking than patients who participate. By not participating, you can lose out on learning how to control your cravings and have a greater quality of life.

The gain frame read as follows:

The good news is, quitting, or even reducing the number of cigarettes you smoke each day, could be much easier with the support of our study. In fact, the Smoke Free Support Study has shown that patients who participated were 3 times more likely to successfully quit smoking than the average patient. By participating, you can benefit from learning how to control your cravings and have a greater quality of life.

### Quantitative Measures

#### Sociodemographics

The following sociodemographic characteristics were measured: gender (male, female, transgender, gender nonconforming, or other), race (American Indian or Alaskan Native, Asian, Black or African American, Native Hawaiian or Pacific Islander, White, or other), ethnicity (Hispanic and Latino or not Hispanic and Latino), age (in years), household income (≥US $40,000), and highest level of education (after high school education or above).

#### Cancer Characteristics

The type of cancer diagnosis (prostate, lung, breast, pancreas, skin, stomach, gynecological, colorectal, and other) and months since diagnosis (>6 months, 7-12 months, or 13-24 months) were assessed.

#### Smoking Characteristics

The following smoking characteristics were assessed: the number of years smoked or how long the participant had smoked cigarettes in years, Heaviness of Smoking Index measured across 2 items or how many cigarettes the participant smoked per day, how soon after the waking up does the participant smoke (within 5 minutes, 6-30 minutes, 31-60 minutes, and after 60 minutes) [27], and smoking urge or how much of the time the participant felt the urge to smoke in the past 24 hours (all the time, almost all the time, much of the time, some of the time, a little of the time, or not at all). Participants’ attitudes toward quitting were measured using the 4 dimensions previously used by the authors (blinded for review): importance or how important it was that the participant quit smoking, ranging from 0 (not important at all) to 10 (very important); confidence or how confident the participant was they could quit smoking, ranging from 0 (not confident at all) to 10 (very confident); how much quitting smoking would reduce the participant’s chances of developing cancer, ranging from 0 (not at all) to 10 (very much); and Biener Contemplation ladder for stage of motivation to quit smoking (“I have decided to continue smoking”; “I do not think about quitting smoking”; “I rarely think about quitting and have no plans to quit”; “I sometimes think about quitting but I have no plans yet”; “I often think about quitting but I have no plans yet”; “I plan to quit smoking in the next 6 months”; “I plan to quit smoking in the next 30 days”; “I have begun to make changes in my smoking”; “I have made changes in my smoking but I need to keep working at it”; and “I have already quit smoking”) [28].

### Message Evaluation

#### Message Relevance

Perceived message relevance was measured using 2 items from the Perceived Message Relevance Scale [29,30]. Items measured how personalized or customized the stimuli were (eg, “The video seemed to be made personally for me”). Items were measured on a 5-point Likert scale, with response categories ranging from 1 (strongly disagree) to 5 (strongly agree; α=.79; mean 4.26, SD 0.91).

#### Message Credibility

Perceptions of informational credibility were measured using items from Appelman and Sundar [31] and assessed participants’ perceptions that the video was accurate, credible, and believable. The 3 items (eg, “The information discussed in the video is accurate”) were rated on a 5-point Likert scale, with response categories ranging from 1 (strongly disagree) to 5 (strongly agree; α=.81; mean 4.34, SD 0.68).

#### Message Clarity

Perceptions of message clarity were adapted from Cacioppo et al [32] and measured the extent to which participants perceived the content of the video to be clear, which was measured on a 1-item, 5-point Likert scale, with responses ranging from 1 (strongly disagree) to 5 (strongly agree). The item stated, “The content in the video is clearly explained” (mean 4.34, SD 0.68).

### Message Effectiveness

#### Improved Perceptions

Improved perceptions of smoking cessation research were measured using a 1-item, investigator-developed measure on a 5-point Likert scale, with response categories ranging from 1 (strongly disagree) to 5 (strongly agree). The item stated, “The video improved my view of smoking cessation research” (mean 4.08, SD 0.85).

#### Information Seeking

Information seeking about participation in a smoking cessation study was measured using a 1-item investigator-developed measure on a 5-point Likert scale, with response categories ranging from 1 (strongly disagree) to 5 (strongly agree). The item stated, “I am interested in more information about enrolling in a smoking cessation study” (mean 4.74, SD 1.48).

#### Informed Decision-making

Informed decision-making about participation in a smoking cessation study was measured using a 1-item, investigator-developed measure on a 5-point Likert scale, with response categories ranging from 1 (strongly disagree) to 5 (strongly agree). The item stated, “With this video, I believe I can make an informed decision on participation in a smoking cessation study” (mean 4.15, SD 0.77).

### Message Outcome: Intent to Talk to a Physician About Participating

The intent to participate in a smoking cessation study was measured using a 1-item, investigator-developed measure on a 5-point Likert scale, with response categories ranging from 1 (strongly disagree) to 5 (strongly agree). The item stated, “I intend to talk to my doctor about enrolling in a smoking cessation study” (mean 4.28, SD 1.86).

### Qualitative Measure: Open-Text Responses

Participants provided open-text feedback on the video by responding to the following prompt: “In the space below, please tell us what you thought about the video you just saw.”

### Statistical Analyses

#### Phase 1: Message Design Experiment

Summary statistics were used to report means with SDs for continuous variables and frequencies with percentages for categorical variables. Message evaluation, message effectiveness, and message outcome variables were compared using ANOVA to examine the main effect of the 3 message factors compared with the control and within-message factor levels. This study was not powered for interactions among the 3 factors. To determine the predictors of intent to talk to a physician about participating in a smoking cessation study, univariate analyses were conducted to determine the relationships among participants’ sociodemographic characteristics, cancer characteristics, smoking characteristics, and message evaluation and effectiveness measures of intent to participate. Variables with *P*≤.10 were included in the multivariable model, as well as message factors that were shown to have a main effect on intent. A generalized linear model was used to identify significant predictors in the multivariable model with a 2-sided significance level of .05. All analyses were conducted using IBM SPSS Statistics for Mac software (version 26).

#### Phase 2: Open-Text Response Analysis

Open-text data were analyzed using 2 software packages: Leximancer and Linguistic Inquiry Word Count (LIWC). First, Leximancer was used as a text-mining software to generate a concept map. Leximancer uses machine learning to generate a codebook, identify related keywords to form concepts, and then map the relationships between concepts based on the level of association between words or phrases. Second, Leximancer was used to conduct automated thematic analysis. Themes are generated when clusters of concepts are linked and can encapsulate broader phenomena. Themes are then given *hits* to determine their frequency or salience in the text. Within the concept map, the size of the theme is directly proportional to its frequency in the data. To form the map, themes are linked together with pathways that help to provide insight into whether the themes are connected. Previous studies have used Leximancer as a tool to triangulate qualitative data [33], analyze a large corpus of open-text data to identify markers of risk communication [34], and evaluate the mechanisms by which tailoring risk messages to promote colorectal cancer messages may be effective [35].

For this study, participant responses were uploaded to Leximancer to generate a preliminary concept map to understand the primary grouping and frequencies of the concepts. The experimental conditions were not separated and used to generate independent concept maps because of sample size limitations. Upon reviewing the preliminary concept map, study team members (JN, CS, and LB) identified and then grouped similar words (eg, *quits*, *quitting*, and *quit*) to refine the autogenerated concepts and create the final concept map. Leximancer used the cleaned data to generate the best-fitting quotes for each theme, and this output was analyzed by the study team members to generate a definition for each theme and pick an exemplary quote. To ensure rigor within this iterative process, the study used the constant comparative method [14]; that is, 2 coders (CS and LB) independently reviewed the Leximancer output and then reviewed together afterward to discuss reflections. These 2 members then brought their impressions and any discrepancies to a 3-member consensus group (JN, CS, and LB), which met weekly. A senior investigator and expert in qualitative methods (EP) then provided a process evaluation and a final review of the concepts.

LIWC is a textual analysis software that compares text-based data with a group of built-in dictionaries. The LIWC dictionaries are summary language variables and specific language variables. LIWC has been used to identify linguistic markers or conduct sentiment analysis within diverse open-text data, interpersonal or web-based medical communication contexts [36], and extensively within contexts related to cancer [37-41]. This study used LIWC to analyze word count and selected 4 summary language variables (analytical thinking, clout, authenticity, and emotional tone), presence of relevant psychological variables (overall affect, positive emotion, and negative emotion), and drives and needs variables (reward and risk). The summary language variables were calculated and converted to percentiles based on standardized scores from large comparison samples, whereas the specific language variables were calculated as a percentage of the total words used in the given language sample. ANOVA was used to examine the main effect of the 3 message factors compared with the control and within-message factor levels across summary and specific language variables.

## Results

### Participant Characteristics

**Figure 1:**
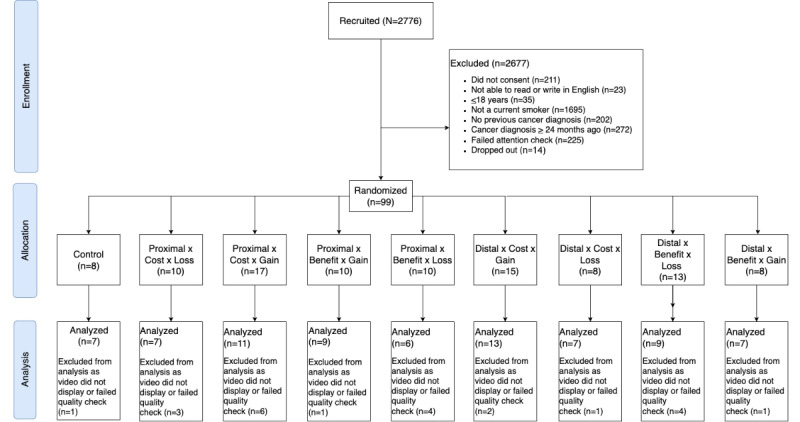
CONSORT (Consolidated Standards of Reporting Trials) flow diagram.

A total of 99 participants were recruited and consented to participate in the study (Figure 1). Of the 99 participants, 22 (22%) participants were excluded from the final sample as they indicated that the video did not display (7/99, 7%), had a benign tumor (1/99, 1%), or failed the study attention check (15/99, 15%). Thus, 76 participants were included in the final analysis. Table 2 reports the characteristics of the 76 participants, who had a mean age of 53.4 (SD 1.6) years, were male (42/76, 55%), were predominantly White (65/76, 86%), and completed formal education after high school (62/76, 82%). Almost all participants had health insurance (73/76, 96%), and most had a household income >US $40,000 (56/76, 73.7%). The most frequently reported cancers were of the skin (23/76, 30%) and breast (10/76, 13%), with over one-third of the participants diagnosed with cancer in the past 6 months (29/76, 38%). Participants reported a lifetime of nicotine use through the number of years in which they smoked cigarettes (mean 28.93, SD 16.41), as well as a current dependence on cigarettes smoked per day (mean 11.84, SD 7.91) and time to first cigarette (<30 minutes; 24/76, 31.6%).

**Table 2:** Participant characteristics compared across 9 conditions (N=76).

Participant characteristics					Total		Control		Proximal, cost, loss		Proximal, cost, gain		Proximal, benefit, gain		Proximal, benefit, loss		Distal, cost, gain		Distal, cost, loss		Distal, benefit, loss		Distal, benefit, gain
Age (years), mean (SD)					53.4 (1.6)		57.6 (17.8)		59.7 (11.9)		48.5 (16.9)		54.8 (11.3)		63.8 (9.2)		52.9 (15.1)		49.1 (12.6)		52.9 (12.4)		45.4 (15.0)
**Gender, n (%)**																							
	Male			42 (55)		5 (71)		4 (57)		7 (64)		1 (11)		3 (50)		9 (69)		4 (57)		4 (44)		5 (71)	
	Female			34 (45)		2 (29)		3 (43)		4 (36)		8 (89)		3 (50)		4 (31)		3 (43)		5 (56)		2 (29)	
**Race, n (%)**																							
	White			65 (86)		6 (86)		5 (71)		9 (82)		7 (77.8)		6 (100)		13 (100)		6 (86)		8 (89)		5 (71)	
	Non-White			11 (15)		1 (14)		2 (29)		2 (18)		2 (22)		0 (0)		0 (0)		1 (14)		1 (11)		2 (29)	
**Ethnicity, n (%)**																							
	Hispanic			7 (9)		0 (0)		1 (14)		0 (0)		0 (0)		1 (17)		1 (8)		2 (29)		0 (0)		2 (29)	
**Education, n (%)**																							
	After high school education			62 (82)		6 (96)		4 (57)		11 (100)		6 (67)		4 (67)		12 (92)		7 (100)		7 (78)		5 (71)	
**Health insurance, n (%)**																							
	Insured			73 (96)		6 (86)		7 (100)		11 (100)		8 (89)		6 (100)		13 (100)		6 (86)		9 (100)		7 (100)	
**Income (US $), n (%)**																							
	≥40,000			56 (74)		6 (86)		4 (57)		9 (82)		5 (56)		3 (50)		8 (62)		7 (100)		8 (89)		6 (86)	
**Time frame of cancer diagnosis (months), n (%)**																							
	<6			29 (38)		2 (29)		5 (71)		4 (36)		3 (33)		2 (33)		5 (39)		2 (29)		4 (44)		2 (29)	
	7-12			28 (37)		3 (43)		1 (1)		3 (27)		3 (33)		2 (33)		5 (39)		4 (57)		3 (33)		4 (57)	
	13-24			19 (25)		2 (29)		1 (14)		4 (36)		3 (33)		2 (33)		3 (23)		1 (14)		2 (22)		1 (14)	
**Cancer screening history, n (%)**																							
	Prostate			7 (9)		0 (0)		1 (14)		4 (36)		1 (11)		0 (0)		0 (0)		0 (0)		0 (0)		1 (14)	
	Lung			5 (7)		0 (0)		0 (0)		1 (9)		0 (0)		0 (0)		1 (8)		2 (29)		0 (0)		1 (14)	
	Breast			10 (13)		1 (14)		0 (0)		2 (18)		3 (33)		1 (17)		0 (0)		1 (14)		2 (22)		0 (0)	
	Pancreatic			3 ()		0 (0)		2 (29)		0 (0)		0 (0)		0 (0)		1 (8)		0 (0)		0 (0)		0 (0)	
	Skin			23 (30)		1 (14)		3 (43)		2 (18)		1 (11)		2 (33)		4 (31)		2 (29)		5 (56)		3 (43)	
	Stomach			3 (4)		1 (14)		0 (0)		1 (9)		0 (0)		0 (0)		0 (0)		0 (0)		0 ()		1 (14)	
	Gynecological			7 (9)		0 (0)		0 (0)		1 (9)		2 (22)		1 (17)		1 (8)		1 (14)		1 (11)		0 (0)	
	Colorectal			7 (9)		1 (14)		1 (14)		0 (0)		0 (0)		0 (0)		2 (15)		1 (14)		1 (11)		1 (14)	
	Other			10 (13)		2 (29)		0 (0)		0 (0)		2 (22)		2 (33)		4 (31)		0 (0)		0 (0)		0 (0)	
	Never screened for any test			1 (1)		1 (14)		0 (0)		0 (0)		0 (0)		0 (0)		0 (0)		0 (0)		0 (0)		0 (0)	
**Smoking characteristics, n (%)**																							
	**eHealth literacy**																						
		Values, mean (SD)		3.90 (0.65)		3.73 (0.76)		3.93 (0.68)		3.73 (0.78)		3.75 (0.49)		4.31 (0.39)		3.78 (0.71)		3.88 (0.77)		4.14 (0.34)		4.09 (0.77)	
		Values, range		2.0-5.0		2.4-4.5		2.5-4.5		2.3-4.9		2.9-4.4		3.8-4.8		2.0-4.5		2.8-5.0		3.8-4.8		2.6-5.0	
	**Years smoked**																						
			Values, mean (SD)	28.93 (16.41)		33.29 (22.49)		27.86 (15.77)		24.00 (19.69)		33.67 (8.65)		38.00 (15.79)		32.46 (16.25)		23.71 (14.87)		25.67 (14.14)		22.43 (18.28)	
			Values, range	2-57		2-54		10-50		2-54		17-42		18-55		10-55		5-45		3-51		2-57	
	**Cigarettes smoked per day**																						
		Values, mean (SD)		11.84 (7.91)		13.57 (10.53)		6.86 (3.67)		12.09 (4.89)		9.67 (5.92)		15.67 (7.53)		12.92 (7.92)		9.00 (8.64)		13.25 (11.30)		13.43 (10.05)	
		Values, range		0-35		0-30		3-12		5-20		0-18		9-30		0-30		0-25		0-35		2-30	
**Minutes to first cigarette, n (%)**																							
	>30			24 (32)		2 (29)		2 (29)		4 (363)		5 (56)		0 (0)		3 (23)		3 (43)		4 (44)		1 (14)	
	<30			51 (67)		5 (71)		5 (71)		7 (64)		4 (44)		6 (100)		10 (77)		4 (57)		4 (44)		6 (86)	
**Quit importance**																							
	Values, mean (SD)			8.28 (1.86)		7.71 (2.36)		9.43 (0.79)		8.60 (1.58)		8.00 (2.18)		7.00 (2.97)		8.31 (1.49)		8.00 (2.38)		8.44 (1.51)		8.71 (1.38)	
	Values, range			3-10		5-10		8-10		6-10		4-10		3-10		6-10		3-10		5-10		7-10	
**Quit confidence**																							
	Values, mean (SD)			7.07 (2.41)		7.00 (1.83)		8.43 (1.51)		6.73 (2.57)		7.33 (2.06)		6.83 (2.48)		5.38 (3.07)		8.00 (0.82)		7.33 (3.00)		8.00 (1.73)	
	Values, range			1-10		5-10		6-10		2-10		3-10		3-10		1-10		7-9		2-10		5-10	
**Benefits of quitting to reduce cancer risk**																							
	Values, mean (SD)			4.96 (2.73)		4.86 (3.19)		5.57 (2.23)		3.45 (2.21)		6.22 (2.95)		5.17 (1.94)		5.08 (3.15)		4.29 (2.75)		5.67 (3.43)		4.57 (1.90)	
	Values, range			1-10		1-10		1-8		1-6		1-10		3-8		1-10		2-10		1-10		1-7	
**Intention to quit smoking**																							
	Values, mean (SD)			2.94 (1.08)		3.17 (0.75)		2.67 (1.63)		2.64 (1.21)		2.75 (1.16)		2.83 (0.75)		2.85 (0.90)		3.17 (1.17)		3.14 (1.21)		3.57 (0.98)	
	Values, range			1-5		2-4		1-5		1-5		2-5		2-4		2-5		1-4		1-5		2-5	

### Message Design Experiment

#### Message Evaluation, Message Effectiveness, and Message Outcome

First, the message frames were compared with those of the control (Table 3). The control condition reported lower mean values for almost every measure; however, there were no statistically significant differences. Next, message frames were compared within the factors (eg, proximal vs distal). Across message evaluation measures, all messages performed equally well across the perceived message relevance, credibility, and clarity measures. In the message effectiveness measures, participants who received the proximal threat message frame reported a significantly greater interest in talking to their physician about participating in a smoking cessation research study when compared with the distal frame (*F*_1, 67_=4.49; mean distal 4.83, SD 1.61, v. mean proximal 5.55, SD 1.15; *P*=.04). There were no statistically significant differences between the cost of smoking frame versus the benefits of quitting frame. However, participants who received the loss of not participating message frame reported significantly improved perceptions of smoking cessation research (*F*_1, 67_=4.20; mean gain 3.98, SD 0.83, vs mean loss 4.38, SD 0.78; *P*=.04). In the message outcome measure, participants in the costs of not quitting message frame reported significantly greater intention to speak to their physician about enrolling (*F*_1, 67_=4.47; mean cost 5.13, SD 1.70) vs mean benefit 4.23, SD 1.86; *P*=.04).

**Table 3:** Main effects for message evaluation, message effectiveness, and message intent for each message factor.

Message factor		Control, mean (SD)	Distal, mean (SD)	Proximal, mean (SD)	*P* value^a^	Cost, mean (SD)	Benefit, mean (SD)	*P* value^a^	Gain, mean (SD)	Loss, mean (SD)	*P* value^a^
**Message evaluation**											
	Message relevance	3.50 (1.29)	3.99 (0.87)	4.18 (0.84)	.34	4.16 (0.81)	3.98 (0.90)	.40	4.06 (0.82)	4.10 (0.91)	.85
	Message credibility	3.95 (0.78)	4.46 (0.68)	4.29 (0.63)	.29	4.38 (0.62)	4.39 (0.71)	.95	4.33 (0.68)	4.45 (0.64)	.48
	Message clarity	4.43 (0.68)	4.64 (0.64)	4.64 (0.64)	.99	4.58 (0.68)	4.71 (0.53)	.39	4.58 (0.64)	4.72 (0.59)	.33
**Message effectiveness**											
	Improved perceptions about smoking cessation research	3.43 (0.79)	4.14 (0.87)	4.15 (0.80)	.95	4.18 (0.80)	4.10 (0.87)	.67	3.98 (0.83)^b^	4.38 (0.78)^b^	.04^b^
	Informed decision-making about participating in a smoking cessation research study	4.71 (0.49)	4.22 (0.83)	4.36 (0.74)	.46	4.29 (0.80)	4.29 (0.78)	1.0	4.30 (0.72)	4.28 (0.88)	.90
	Interest in further information about participating in a smoking cessation research study	4.14 (1.57)	4.83 (1.61)^b^	5.55 (1.15)^b^	.04^b^	5.18 (1.37)	5.16 (1.56)	.95	5.15 (1.49)	5.21 (1.40)	.87
**Message outcome**											
	Intent to talk to a physician about participating in a smoking cessation research study	4.43 (2.07)	4.78 (2.00)	4.67 (1.63)	.80	5.13 (1.70)^b^	4.23 (1.86)^b^	.04^b^	4.60 (1.89)	4.90 (1.72)	.51

^a^*P* values are for comparison of main effects between message factor levels.

^b^*P* values <.05

#### Predictors of Intention to Speak to a Physician About Enrolling in a Smoking Cessation Study

Participants’ sociodemographic characteristics, smoking and cancer characteristics, and message evaluation and message effectiveness predictors were explored to determine their association with the message outcome—their intention to speak to their physician about enrolling in a smoking cessation study. Univariate predictors that were associated with intent to speak to a physician included younger age (*P*=.06), male gender (*P*=.003), greater urge to smoke (*P*=.02), greater importance of quitting (*P*=.002), greater confidence in quitting (*P*=.04), greater perceived message relevance (*P*<.001), and improved perceptions about smoking cessation research (*P*=.002). In the multivariable model (Table 4), univariate predictors and cost versus benefit message factors were included because of the significant main effects discussed previously. The overall model was significant (*F*_8,55_=6.33; *P*<.001), explaining 47.9% of the variance in the intention to speak to a physician about participating. Within the model, male participants were significantly less likely (β=−.24, *P*=.02), whereas participants who reported greater baseline importance of quitting (β=.24, *P*=.046) and perceived the message as relevant to their situation (β=.37, *P*=.004) were significantly more likely to intend to speak to their physician about participating in the study. With the inclusion of the study covariates, the main effect of the cost versus benefit message factor was no longer statistically significant (β=−.17, *P*=.12).

**Table 4:** Multivariable predictors of intent to speak to a physician about enrolling in a smoking cessation research study.

Predictor	β	SE	*t* test (*df*=8)	*P* value	95% CI
Age (years)	−.01	0.01	−0.88	.38	−0.03 to 0.01
Gender (male)	−.90	0.32	−2.85	.006	−1.54 to −0.27
Urge to smoke	.30	0.15	1.94	.06	−0.01 to 0.61
Quit importance	.17	0.09	1.82	.07	−0.02 to 0.36
Quit confidence	.09	0.08	1.11	.27	−0.07 to 0.24
Improved perceptions about smoking cessation research	−.09	0.22	−0.40	.69	−0.52, 0.35
Message relevance	.77	0.22	3.58	.001	0.34 to 1.20
Cost versus benefit condition (cost as referent)	−.29	0.32	−0.91	.37	−0.93 to 0.35

### Open-Text Analysis

#### Leximancer

**Figure 2:**
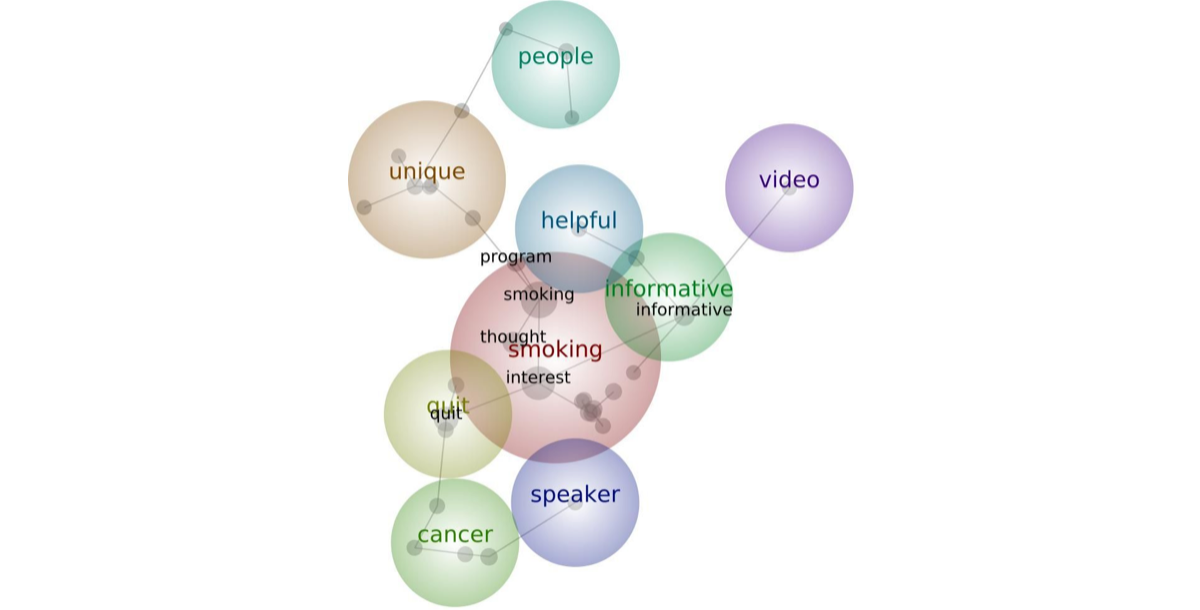
Leximancer-generated concept map detailing participant responses when asked to evaluate the video.

The Leximancer analysis resulted in 8 main themes that emerged from the open-text responses to the participants’ video evaluations. The themes, operational definitions, exemplary quotes, and experimental conditions of the participants are detailed in Table 5. The concept map (Figure 2) visually displays the connectedness of the themes and where thematic bubbles overlap, indicating that sentiments expressed in each concept are not mutually exclusive. There were 3 distinct paths on the concept map, all of which branched from the smoking theme. The first pathway, which links *informative* to *helpful* to *video*, is a cognitive evaluation of the videos and an acknowledgment that their primary function was to inform about a trial that connects smokers to cessation resources. The second pathway, comprising *unique* and *people*, highlights the connection between the type of cessation resources and the person offering those resources (an oncologist) as either unique or not unique. The third pathway, from *quit* to *cancer* to *speaker*, highlights the teachable moment context in which the trial is offered. The participants connected quitting with their treatment and cancer outcomes.

**Table 5:** Selection of an exemplary quote for each theme, along with the frequency of hits for each theme and condition for each quote (N=111 hits).

Theme	Theme definition	Hits of a theme among responses, n (%)	Quote	Participant condition
Smoking behavior and perceptions	Participants’ perceptions about their smoking habits	38 (34.2)	“[The speaker] got me to thinking about my smoking habits, even though I only smoke 4 cigarettes per day.”	Proximal, cost, loss
Motivation or readiness to quit	Participants’ interest, motivation, and readiness to quit smoking	18 (16.2)	“It gave me different options to quit smoking. I really do want to quit, but I don’t think I can.”	Control
How informative the video was	Participants’ explanations of the video being informative	17 (15.3)	“It was very informative and interesting. Being honest about smoking will help in heath related issues”	Distal, benefit, gain
Cancer diagnosis	Participants’ framing of the aspects of the video with regard to their cancer diagnosis	9 (8.1)	“The speaker brought up some good points, such as lower energy levels after being diagnosed with cancer. My energy level hasn’t regained to where I want it to be since prostate surgery.”	Proximal, cost, loss
How relevant the video was	Participants’ descriptions of the video as beneficial or not for people like them	8 (7.2)	“I felt that by joining the study I could get the help I need to stop smoking”	Distal, benefit, gain
Evaluation of the tobacco treatment services offered in the study	Participants’ overall perception of the uniqueness of the program, both positive and negative	7 (6.3)	“A generous offer to participate but nothing presented was unique in that all outlined methods of smoking cessation therapy are already readily available.”	Distal, benefit, loss
Overall opinion of the video	Participants’ overall opinion of the video	6 (5.4)	“It was a very informative and interesting video. I enjoyed watching it.”	Proximal, benefit, loss
Evaluation of the speaker in the video	Participants’ reactions and feedback regarding the video speaker	2 (1.8)	“The speaker was very professional, and not scolding or condescending.”	Distal, cost, loss

#### Theme 1: Smoking Behavior and Perceptions

Smoking behavior was the most common theme, accounting for 34.2% (38/111) of the total hits. The participants reflected on their smoking habits and how they often functioned as a stress management tool. One of the participants indicated the following:

I’ve made three serious attempts and numerous casual attempts at quitting. I will try again this year, but my failure has always been [the] use of smoking as a coping mechanism for stress

distal, cost, gain

Another stated the following:

I’ve reduced the number of cigarettes, but have found no other mechanism for coping with stress despite attempts

proximal, cost, loss

Others revealed that the video prompted self-reflection on the need to address their smoking habits:

She got me to thinking about my smoking habits, even though I only smoke 4 cigarettes per day

proximal, cost, loss

#### Theme 2: Motivation or Readiness to Quit

According to the concept map, the quitting theme understandably overlapped with the smoking theme. However, unique instances of participants’ motivation and hope to quit successfully after previous failed attempts were also identified. One of the participants highlighted the following:

I’ve tried many times and different ways to quit in the past with no success. Maybe one of these ways will help

control

Another reflected on a broader message of hope, potentially resulting from learning about the success rates of treatment discussed in the video:

I thought it gave me hope to quit smoking

proximal, cost, gain

However, others mentioned that learning about new treatment options does not necessarily translate to greater self-efficacy to quit by saying the following:

It gave me different options to quit smoking. I really do want to quit, but I don’t think I can

control

#### Theme 3: How Informative the Video Was

Participant responses categorized under this theme primarily comprised explaining the usefulness of the information in the video. One of the participants commented the following:

It was very informative and interesting. Being honest about smoking will help in [health] related issues

distal, benefit, gain

Other participants reported similar views and added that the videos were honest as well as helpful:

I thought it was very well thought out and honest. Also seemed very helpful for people like me

proximal, cost, gain

#### Theme 4: Cancer Diagnosis

Responses within this theme were related to smoking cessation in the context of personal cancer diagnoses. Comments reflected a diagnosis acting as a teachable moment and motivating quit attempts, albeit not always successfully:

It sounds interesting. I have tried numerous times to quit even though I have been diagnosed with cancer

distal, cost, gain

Some responses demonstrated that the participant had internalized the risk message frame they received and identified with the negative consequences of continued smoking after a cancer diagnosis:

The speaker brought up some good points, such as lower energy levels after being diagnosed with cancer. My energy level hasn’t regained to where I want it to be since prostate surgery

proximal, cost, loss

#### Theme 5: How Relevant the Video Was

This theme reflected on the personal support structures the trial would provide:

I felt that by joining the study I could get the help I need to stop smoking

distal, benefit, gain

Other responses were as follows:

informative, relatable, held interest and would be a welcome program (support study) in my area

proximal, benefit, loss

#### Theme 6: Evaluation of the Tobacco Treatment Services Offered in the Study

Perspectives on the trial diverged greatly depending upon the participant’s impressions of how unique they felt the resources offered as part of the trial were. For example, one of the participants commented that the study was “extremely unique, valuable, appealing, and potentially lifesaving. Hard to believe program is free, and offers patches to assist in overall probable successful, life changing outcome” (proximal, benefit, loss), whereas another participant commented the following:

a generous offer to participate, but nothing presented was unique in that all outlined methods of smoking cessation therapy are already readily available

distal, benefit, loss

#### Theme 7: Overall Opinion of the Video

This theme was composed of a range of perspectives but broadly discussed the method of presenting trial information digitally. The participants commented that “it was a very informative and interesting video. I enjoyed watching it” (proximal, benefit, loss).

#### Theme 8: Evaluation of the Speaker in the Video

Participants’ responses under this theme evaluated the speaker within the video, focusing on the oncologist’s tone and demeanor when presenting the importance of quitting after a diagnosis. One of the participants commented the following:

the speaker was very professional, and not scolding or condescending

distal, cost, loss

Another participant similarly discussed the following:

I thought the speaker was very informative

proximal, cost, loss

#### LIWC Analysis

Within the LIWC analyses, there were no significant differences between control and message factors or within message factor levels (eg, distal vs proximal) for the 5 summary variables (word count, analytic thinking, clout, authentic, and emotional tone). Next, comparisons were made across psychological processes (affect, positive emotion, and negative emotion) and drivers and needs (reward and risk). Compared with the control group (mean control 28.32, SD 35.82), participants in both the distal and proximal message frames used linguistic markers that reflected statistically significant lower levels of affect (*F*_2,72_=3.13; mean distal 17.20, SD 22.04; mean proximal 9.54, SD 10.17; *P*=.05), as did the gain and loss message frames (*F*_2,72_=3.47; mean gain 17.16, SD 20.51, mean loss 8.55, SD 11.34; *P*=.04). Compared with the control (mean control 15.02, SD 37.52), the distal and proximal (*F*_2,72_=5.70; mean distal 0.05, SD, 0.30, mean proximal 0.71, SD 2.49; *P*=.005), cost and benefit (*F*_2,72_=5.68; mean cost 0.20, SD 0.95, mean benefit 0.57, SD 2.41; *P*=.005), and gain and loss (*F*_2,72_=5.69; mean gain 0.60, SD 2.30, mean loss 0.06, SD 0.32; *P*=.005) message frames reported significantly lower levels of negative emotions.

Within message factor levels, participants who saw the distal message used linguistic markers that reflected significantly greater positive emotions than participants who saw the proximal message (*F*_1,66_=3.87; mean distal 17.16, SD 22.07 vs mean proximal 8.84, SD 10.40; *P*=.05). However, participants who watched the proximal message used linguistic markers that reflected a significantly greater risk than those who saw the distal message (*F*_1,66_=4.13; mean distal 0.00, SD 0.00 vs mean proximal 0.98, SD 2.85; *P*=.05). There were no differences in the cost versus benefit message frames. Within the gain versus loss message frame, participants who watched the gain message used linguistic markers that reflected significantly greater affect (*F*_1,66_=4.16; mean gain 17.16, SD 20.51 vs mean loss 8.55, SD 11.34; *P*=.05).

## Discussion

### Principal Findings

Access to evidence-based tobacco treatment among smokers with recent diagnoses remains a priority. A method of increasing access is participation in tobacco treatment trials. Although accrual rates remain suboptimal, targeted digital outreach through short recruitment videos may offer promise but has not been assessed specifically among patients newly diagnosed with cancer. Multimethod approaches are required to optimize the content of these videos. Therefore, this pilot factorial randomized controlled trial explored which message frames were most effective for a video to recruit smokers with a recent cancer diagnosis for a tobacco treatment trial.

In phase 1, a message design experiment assessed 3 message frames: message evaluation, effectiveness, and outcome measures. For the primary outcome, the costs of not quitting the frame increased the intent to speak to a physician about participating in a cessation study significantly when compared with the benefits of the quitting frame. This is an important finding that does not align with most of the literature, in which gain-framed messages have been predominantly demonstrated to be more effective at promoting cessation [19,20]. However, when cancer treatment outcomes are central, highlighting the negative side effects of continued smoking, including psychological (ie, an increase in anxiety and stress) and physiological (ie, a decrease in energy) effects, motivation to avoid these side effects may be a stronger mechanism for the uptake of cessation resources. However, it should be noted that in the multivariable model, this effect did not remain significant. As with our previous work, perceptions of the relevance of the message, irrespective of what message frames were used, were much more strongly predictive of intent to want to participate (authors blinded for review). Information processing theories (eg, the Elaboration Likelihood Model [42]) explicate those greater perceptions of message relevance are associated with deeper systematic processing, which elicits greater perceptions of argument strength and motivation to adhere to a message’s call to action (ie, participating in a tobacco treatment trial). Interestingly, message relevance was even more strongly associated with intent to participate than baseline quit importance or confidence. This suggests that identification with the content and context in which a recruitment message is presented may be a more influential mechanism than pre-existing cessation attitudes.

Participants who received the proximal message frame (vs the distal frame) were more likely to report a greater interest in seeking information about participating in a cessation study.

Specifically, the proximal message frame used (1) social norms (eg, “Every day, patients with cancer...”) and (2) reduced psychological distance between smoking and inferior treatment outcomes (eg, “may keep growing and you may be less likely to respond to your treatment”). Existing models (eg, the Planned Risk Information Seeking Model [43,44]) indicate that greater perception of individual risk for a disease or adverse outcome is predictive of greater information-seeking intentions. However, motivating intentions through increased risk perception among smokers may be difficult. Previous studies have demonstrated that risk communication interventions for individuals who have received threat-based messages about behavior over extended periods (eg, heavy smokers) may have a limited effect [45,46]. By focusing on cancer treatment efficacy rather than repeating the common negative physiological effects of smoking, the risk message frame seemed more successful in increasing seeking intention.

Participants who received losses from the nonparticipating frame (vs gains from participating) were more likely to report that the recruitment video positively changed their perspective on smoking cessation research. Patients with cancer may be more sensitive to losses as they have likely recently experienced other *losses*, such as control of their health, their day-to-day routine, or even a loss of their old identity, and now see themselves as patients or survivors of cancer. The prospect theory explicates that losses can loom larger than commensurate gains and losing out on an opportunity framed to have short-term self-efficacy (ie, more difficult without the resources provided through the trial) and long-term response efficacy outcomes (ie, 3 times less likely to stop smoking on own) may have been more compelling when describing the advantages of participating in tobacco treatment trials, especially for patients with cancer.

In phase 2, the multimethod evaluation provided a further understanding of how the recruitment videos were appraised. In the Leximancer analysis, participants commonly made statements that were thematically associated with smoking and quitting. Smoking was discussed as a coping mechanism for stress, although stress was not specifically discussed as a result of a diagnosis. Some participants reflected on the need to address their smoking habits, admitting that despite their recent diagnosis, they continued to smoke and that tobacco treatment was necessary. The Leximancer analysis did not compare data by message frame; however, some responses highlighted that the participants reflected on the information provided in at least one of the message frames to which they were randomized. For example, participants were able to identify with the risk messaging, make a connection to their own cancer journey, and mention how they felt because of continuing to smoke after the diagnosis (eg, how severe their side effects were during treatment).

The LIWC analysis compared the linguistic differences in the open-text data between the message factors to the control condition and within the message factor levels. The findings demonstrated that participants in the control condition used language to describe the video with significantly greater levels of negative emotion than those in the intervention conditions. This finding suggests that the information included in any of the message frames, irrespective of the frame, reduced negative emotions. Although the control condition functioned as a kernel message and encompassed all the necessary trial information, the message frames provided intrinsic and extrinsic motivations to want to participate and likely reduced psychological reactance when presented with the trial. Within the message factor levels, a noteworthy finding was that participants who viewed the proximal message frame had linguistic markers that reflected a significantly greater internalized risk than participants who viewed the distal message. This finding is consistent with the psychological distance of risk explicated within the construal-level theory, which suggests that individuals will construe future events more concretely if they are temporally more proximal [17]. As the short-term risk associated with a current diagnosis (eg, worse treatment outcomes) is temporally and psychologically more concrete, participants used language to describe the video that incorporated more linguistic markers of proximal risk. Measuring risk internalization in this way is novel but also underscores the challenge of using threat-based messaging to invoke perceptions of risk. This was exemplified in comparison with the distal message frame, in which participants used linguistic markers with more positive emotions, suggesting that risk internalization can create an emotional response if experienced immediately and, thus, more concretely.

### Limitations

This study has a number of strengths, although there are also limitations. First, the recruitment videos promoted a specific cessation trial (ie, Smoke Free Support 2.0), which was not actively available for the enrollment of participants. The findings of this study could have been further tested if participants who had indicated they intended to quit smoking were then directed to n web-based resource that connected them to an active cessation trial in their community (eg, Research Match). Second, the sample was predominantly White and educated and had health insurance. This limits generalizability and does little to address the crucial need to test recruitment videos with underrepresented groups who report greater medical mistrust and lower representation in clinical trials [47]. To address this issue, we are actively conducting multiple studies to develop and disseminate bilingual, culturally tailored recruitment materials to increase the participation of underrepresented groups within a National Cancer Institute–funded tobacco treatment trial. Third, the inclusion criteria (eg, cancer diagnosis) were self-reported rather than verified through an electronic health record, as in the parent trial. Relatedly, we did not collect prognostic measures for the cancer stage. Risk internalization was a key mechanism for understanding the effect of message factors and was a potential confounder. However, we decided against collecting this measure as self-reported prognosis from patients would likely be inaccurate and skewed to a greater perceived likelihood of survival [48,49].

### Implications and Future Research

The primary purpose of this study was to pretest recruitment messages before implementation in the SmokeFree Support 2.0 parent trial, an ongoing nationwide clinical trial across 49 subaffiliates in the National Cancer Institute Oncology Research Program. Findings from this pilot factorial randomized controlled trial identified that message frames that focused on consequences and more immediate outcome expectancies (ie, proximal risks, costs of continued smoking, and the losses of not participating) were the most effective. However, as this study was not powered for interaction effects and the main effects of the message factors were not significant in the multivariable model, a clinical research advisory board discussed whether recruitment messages that used all 3 negative frames would be dissuading for patients so soon after diagnosis. Concerns were also discussed regarding whether clinicians would be comfortable recording and using a script that included multiple negative outcome expectancy frames for patients at their site. As a result, an informed decision was made to implement a recruitment video at sites that included the proximal threat frame but focused on the benefits of quitting and the benefits of participation message frames.

Future studies should first replicate these pilot findings within a clinical sample so as to further explore whether recency and type of diagnosis, as well as stage, affect intention to participate in a tobacco treatment trial. The combination of these factors may result in a greater *teachable moment* (eg, invitation to join a trial the day of a diagnosis compared with 6 months after diagnosis), which may have a meaningful effect on risk internalization and can only be feasibly conducted at a clinic. Furthermore, as perceived message relevancy remained the strongest predictor in the multivariable model, future studies should manipulate other message components to increase perceptions of relevancy. These may include the source (eg, clinician vs patient), medium (eg, text vs video), and the degree to which the content is tailored to each potential participant (eg, tailored to the current motivation to quit and perceived barriers to trial participation).

### Conclusions

Reducing smoking rates among patients with recently diagnosed cancer remains a public health priority. Clinical trials on tobacco treatment can provide timely, evidence-based interventions to facilitate cessation. This study used a novel multimethod approach that leveraged both experimental and open-text data to guide decision-making on how best to design recruitment messages for an ongoing national tobacco treatment trial. The findings indicated that focusing on the negative and more immediate outcomes of not quitting was the most effective. The development and testing of theory-driven and evidence-based recruitment messages should be a key process in all trials seeking to leverage digital outreach to increase accrual rates.

## Appendix

Multimedia Appendix 1 CONSORT-eHEALTH checklist (V 1.6.2).

## Abbreviations

LIWC: Linguistic Inquiry Word Count
