# Supplementary material for: A Multimethod Evaluation of Tobacco Treatment Trial Recruitment Messages for Current Smokers Recently Diagnosed With Cancer: Pilot Factorial Randomized Controlled Trial
Source: JMIR Cancer. 2022 Aug 24;8(3):e37526. doi: 10.2196/37526 (PMC9453579; doi:10.2196/37526)
Supplement: Multimedia Appendix 1 [file cancer_v8i3e37526_app1.pdf]

CONSORT-EHEALTH Checklist V1.6.2 Report	Manuscript Number	37526
**(based on CONSORT-EHEALTH V1.6), available at [http://tinyurl.com/consort-ehealth-v1-6].**		
**Date completed**		
4/25/2021 19:11:51		
**by**		
Jordan NEil		
Increasing smokers' intentions to participate in a cessation study offered at the point of lung screening: Randomized Controlled Trial.		
TITLE		
**1a-i) Identify the mode of delivery in the title**		
"A web-based intervention"		
**1a-ii) Non-web-based components or important co-interventions in title**		
N/A		
**1a-iii) Primary condition or target group in the title**		
"smokers' intentions to participate in a cessation study offered at the point of lung screening"		
ABSTRACT		
**1b-i) Key features/functionalities/components of the intervention and comparator in the METHODS section of the ABSTRACT**		
"Participants were randomized to a 2 (benefits of quitting vs. risks of continuing to smoke at the time of lung screening) x 2 (gains of participating vs. losses of not participating in a cessation study) message design experiment (N=296)"		
**1b-ii) Level of human involvement in the METHODS section of the ABSTRACT**		
N/A		
**1b-iii) Open vs. closed, web-based (self-assessment) vs. face-to-face assessments in the METHODS section of the ABSTRACT**		
"Participants were recruited from a proprietary opt-in online panel company"; "The primary outcome was self-assessed intent to participate in a smoking cessation study"		
**1b-iv) RESULTS section in abstract must contain use data**		
"Two hundred and ninety six participants completed the intervention."		
**1b-v) CONCLUSIONS/DISCUSSION in abstract for negative trials**		
N/A		
INTRODUCTION		
**2a-i) Problem and the type of system/solution**		
"The current study details a formative message design experiment used to determine the most effective message content to include within a recruitment video prior to trial launch"		
**2a-ii) Scientific background, rationale: What is known about the (type of) system**		
"Due to the unique position of promoting a prevention behavior (smoking cessation) within the context of a detection behavior (lung cancer screening), the study was guided by prospect theory. As such, we investigated how best to frame (1) the importance of cessation at the time of lung cancer screening (benefits of quitting vs. risks of continuing to smoke) and (2) information about the study to motivate participation in Screen ASSIST (gains of participating vs. losses from not participating). "		
**Does your paper address CONSORT subitem 2b?**		

"Hypothesis 1 (H1): The risks of continuing to smoke message frame will lead to greater (a) risk perception of developing lung cancer and (b) affective risk response when compared to benefits of quitting smoking message frame."; "H2: The losses of not participating message frame will lead to greater intent to participate in a smoking cessation study compared to gains of participating message frame."; "RQ2: What participant sociodemographic, smoking characteristics, message effectiveness, and lung cancer perception measures are associated with greater intent to participate in a smoking cessation study?<br>RQ3: For message effectiveness and lung cancer perception measures that are associated with intent to participate in a cessation study, are there differences in subgroups defined by participant sociodemographic and smoking characteristics?"		
METHODS		
**3a) CONSORT: Description of trial design (such as parallel, factorial) including allocation ratio**		
"Participants were randomly assigned to receive one of four videos as part of a 2 x 2 factorial design."		
**3b) CONSORT: Important changes to methods after trial commencement (such as eligibility criteria), with reasons**		
N/A		
**3b-i) Bug fixes, Downtimes, Content Changes**		
**4a) CONSORT: Eligibility criteria for participants**		
"A total of 314 participants were recruited for the study but 18 participants indicated the video did not display or they were unable to play it; therefore, responses from 296 participants were included in the final analysis. "		
**4a-i) Computer / Internet literacy**		
NA but eHealth literacy is measured and included in analyses		
**4a-ii) Open vs. closed, web-based vs. face-to-face assessments:**		
"In January 2019, 314 participants were recruited from Qualtrics Panels (Qualtrics, Provo, UT), a proprietary opt-in online panel company, to complete a 20-minute survey."		
**4a-iii) Information giving during recruitment**		
N/A		
**4b) CONSORT: Settings and locations where the data were collected**		
"In January 2019, 314 participants were recruited from Qualtrics Panels (Qualtrics, Provo, UT), a proprietary opt-in online panel company, to complete a 20-minute survey."		
**4b-i) Report if outcomes were (self-)assessed through online questionnaires**		
"All participants completed self-assessment pre-message surveys, and after viewing one of the four videos, participants immediately completed post-message surveys."		
**4b-ii) Report how institutional affiliations are displayed**		
Discussed as limitation "First, the recruitment videos promoted a specific cessation trial (Screen ASSIST), which was offered through a healthcare network in Massachusetts. Past research have discussed a hypothetical study and provided no geographic cues to deter perceptions of access [17,37]. To try and maintain engagement, participants were told before viewing the video that they had similar characteristics to the patients who would be enrolled in Screen ASSIST and that their feedback would improve the video."		
**5) CONSORT: Describe the interventions for each group with sufficient details to allow replication, including how and when they were actually administered**		
**5-i) Mention names, credential, affiliations of the developers, sponsors, and owners**		
N/A		
**5-ii) Describe the history/development process**		
NA this study is described as formative and intervention guided by prospect theory		
**5-iii) Revisions and updating**		
N/A		
**5-iv) Quality assurance methods**		

N/A		
**5-v) Ensure replicability by publishing the source code, and/or providing screenshots/screen-capture video, and/or providing flowcharts of the algorithms used**		
Recruitment videos are currently hosted on an institutional video platform that tracks engagement metrics. As the Screen ASSIST clinical trial is currently still recruiting and using videos, access to videos by non study staff will be provided until trial end.		
**5-vi) Digital preservation**		
N/A no code. Intervention discussed above.		
**5-vii) Access**		
N/A. Discussed as embedded in Qualtrics survey.		
**5-viii) Mode of delivery, features/functionalities/components of the intervention and comparator, and the theoretical framework**		
Stimuli section of the manuscript provides detailed information on intervention.		
**5-ix) Describe use parameters**		
N/A as one-time intervention.		
**5-x) Clarify the level of human involvement**		
N/A		
**5-xi) Report any prompts/reminders used**		
N/A		
**5-xii) Describe any co-interventions (incl. training/support)**		
N/A		
**6a) CONSORT: Completely defined pre-specified primary and secondary outcome measures, including how and when they were assessed**		
Intention to participate is identified as the primary outcome. Exploratory secondary outcomes are detailed.		
**6a-i) Online questionnaires: describe if they were validated for online use and apply CHERRIES items to describe how the questionnaires were designed/deployed**		
N/A		
**6a-ii) Describe whether and how “use” (including intensity of use/dosage) was defined/measured/monitored**		
N/A		
**6a-iii) Describe whether, how, and when qualitative feedback from participants was obtained**		
N/A		
**6b) CONSORT: Any changes to trial outcomes after the trial commenced, with reasons**		
"In January 2019, 314 participants were recruited from Qualtrics Panels (Qualtrics, Provo, UT), a proprietary opt-in online panel company, to complete a 20-minute survey."		
**7a) CONSORT: How sample size was determined**		
**7a-i) Describe whether and how expected attrition was taken into account when calculating the sample size**		
"An a priori power analysis was conducted to ensure the study was powered to detect a medium effect size (Cohen’s d = 0.5) between each message factor level (i.e., BvR and GvL). In the case without message factor interaction, a total sample of 256 participants would permit detection of such a main effect size with 80% power with a two-sided significance level of 0.05. After data collection, interactions between message factors were conducted for each analysis, but none were found to be significant and so are not reported. All analyses were conducted using SAS software, Version 9.4 (SAS Institute, Cary, NC)."		
**7b) CONSORT: When applicable, explanation of any interim analyses and stopping guidelines**		
Intention to participate is identified as the primary outcome. Exploratory secondary outcomes are detailed.		
**8a) CONSORT: Method used to generate the random allocation sequence**		
Randomized through a factorial design.		
**8b) CONSORT: Type of randomisation; details of any restriction (such as blocking and block size)**		
Qualtrics automated randomization.		

**9) CONSORT: Mechanism used to implement the random allocation sequence (such as sequentially numbered containers), describing any steps taken to conceal the sequence until interventions were assigned**		
N/A Qualtrics automated randomization.Qualtrics automated randomization.		
**10) CONSORT: Who generated the random allocation sequence, who enrolled participants, and who assigned participants to interventions**		
N/A Qualtrics automated randomization.		
**11a) CONSORT: Blinding - If done, who was blinded after assignment to interventions (for example, participants, care providers, those assessing outcomes) and how**		
**11a-i) Specify who was blinded, and who wasn't**		
N/A. Study design not appropriate for blinding.		
**11a-ii) Discuss e.g., whether participants knew which intervention was the "intervention of interest" and which one was the "comparator"**		
In the consent form, participants were told the purpose of the study was "Purpose of the Study: Welcome to the United States Healthy Living Survey. We are conducting this study to find out how people aged 55-77 make decisions about their health. By the end of this study, we hope to identify what types of information are most helpful for people to make better decisions about their health behaviors, such as smoking, screening for cancer, etc."<br><br><br>Within the survey, participants were informed: "Thank you for answering questions about your smoking. We would like you to watch a brief video about a smoking cessation study that we will be conducting later this year in Massachusetts. As you have similar characteristics to the patients we hope to enroll in our study, we would like you to review the video and provide us with your feedback.<br><br>Each video takes approximately 3 minutes to watch."		
**11b) CONSORT: If relevant, description of the similarity of interventions**		
N/A.		
**12a) CONSORT: Statistical methods used to compare groups for primary and secondary outcomes**		
"Summary statistics were reported using mean with standard deviation for continuous variables and frequency with percentage for categorical variables. Chi-squared tests were performed to examine the main effect of the two message factors on the completion of watching video and a logistic regression model was used to test the interaction between the two factors. All other manipulation check outcomes, message effectiveness, lung cancer risk perception and intent to participate were compared using analysis of variance (ANOVA) to examine the main effect of the two message factors and the interaction between the two factors."		
**12a-i) Imputation techniques to deal with attrition / missing values**		
N/A as imputation was not conducted		
**12b) CONSORT: Methods for additional analyses, such as subgroup analyses and adjusted analyses**		
"To determine the predictors for intent to participate in a smoking cessation study, univariate analyses were conducted to determine the relationship between participant sociodemographic, smoking characteristics, message effectiveness and lung cancer perception measures and intent to participate. Candidate variables with p values 0.1 or less were included in the variable selection process. The least absolute shrinkage and selection operator (LASSO) approach was used for variable selection in the final multivariable model. The LASSO approach identifies candidate variables and corresponding regression coefficients that lead to a model that minimizes (1) overfitting the number of variables and (2) overestimating of overall model performance, thus, reducing prediction error. A mediation analysis was conducted to determine the direct and indirect effects of message factors on intent to enrol in a cessation trial, guided by statistical principles detailed by VanderWeele [48]. An ANOVA was used to explore differences between subgroups on message effectiveness and lung cancer risk perception variables, in which continuous measures were bifurcated on a mean split (e.g., eHealth literacy) or widely accepted clinical comparisons (e.g., first cigarette within 30 minutes of waking up)."		
RESULTS		
**13a) CONSORT: For each group, the numbers of participants who were randomly assigned, received intended treatment, and were analysed for the primary outcome**		

"A total of 314 participants were recruited for the study but 18 participants indicated the video did not display or they were unable to play it; therefore, responses from 296 participants were included in the final analysis. There were no meaningful differences between the 18 participants and the remaining 296 in participant characteristics (Figure 1 for CONSORT)."		
**13b) CONSORT: For each group, losses and exclusions after randomisation, together with reasons**		
Detailed above.		
**13b-i) Attrition diagram**		
Included in CONSORT diagram		
**14a) CONSORT: Dates defining the periods of recruitment and follow-up**		
"In January 2019".		
**14a-i) Indicate if critical “secular events” fell into the study period**		
**14b) CONSORT: Why the trial ended or was stopped (early)**		
N/A. Trial ended as planned.		
**15) CONSORT: A table showing baseline demographic and clinical characteristics for each group**		
Table 1 displays baseline by condition.		
**15-i) Report demographics associated with digital divide issues**		
eHealth literacy was measured and controlled for in appropriate analyses.		
**16a) CONSORT: For each group, number of participants (denominator) included in each analysis and whether the analysis was by original assigned groups**		
**16-i) Report multiple “denominators” and provide definitions**		
All analyses are presented with a denominator of 296. e.g..."Among the 296, participants had a mean age of 62.9, were predominantly female (66.2%), White (85.8%), did not possess a college degree (74.7%), had health insurance (89.2%), and approximately half reported a household income below $40,000 (47.6%)."		
**16-ii) Primary analysis should be intent-to-treat**		
**17a) CONSORT: For each primary and secondary outcome, results for each group, and the estimated effect size and its precision (such as 95% confidence interval)**		
The primary outcome was non-significant. Mean and standard deviations are reported.		
**17a-i) Presentation of process outcomes such as metrics of use and intensity of use**		
**17b) CONSORT: For binary outcomes, presentation of both absolute and relative effect sizes is recommended**		
N/A		
**18) CONSORT: Results of any other analyses performed, including subgroup analyses and adjusted analyses, distinguishing pre-specified from exploratory**		
Prior to RQ2 and 3, we state ", we propose the following exploratory research questions:"		
**18-i) Subgroup analysis of comparing only users**		
**19) CONSORT: All important harms or unintended effects in each group**		
N/A.		
**19-i) Include privacy breaches, technical problems**		
**19-ii) Include qualitative feedback from participants or observations from staff/researchers**		

DISCUSSION		
**20) CONSORT: Trial limitations, addressing sources of potential bias, imprecision, multiplicity of analyses**		
**20-i) Typical limitations in ehealth trials**		
Yes. Compares Qualtrics sample to other lung cancer screening populations. "When evaluating the implications of these findings, it is important to compare how sample characteristics reflect patient characteristics in other national lung screening programs...."		
**21) CONSORT: Generalisability (external validity, applicability) of the trial findings**		
**21-i) Generalizability to other populations**		
**21-ii) Discuss if there were elements in the RCT that would be different in a routine application setting**		
**22) CONSORT: Interpretation consistent with results, balancing benefits and harms, and considering other relevant evidence**		
**22-i) Restate study questions and summarize the answers suggested by the data, starting with primary outcomes and process outcomes (use)**		
Yes. "There were no significant differences in intent to participate in a cessation research study by message frame." "From the message design experiment, the benefits of quitting frame increased affective risk response when compared to participants who received the risks of continuing to smoke frame."		
**22-ii) Highlight unanswered new questions, suggest future research**		
Other information		
**23) CONSORT: Registration number and name of trial registry**		
N/A. .		
**24) CONSORT: Where the full trial protocol can be accessed, if available**		
N/A		
**25) CONSORT: Sources of funding and other support (such as supply of drugs), role of funders**		
N/A		
**X26-i) Comment on ethics committee approval**		
"Participants received a small compensation for their participation and Institutional Review Board approval was obtained before data collection began (#2018P002035)."		
**x26-ii) Outline informed consent procedures**		
N/A		
**X26-iii) Safety and security procedures**		
**X27-i) State the relation of the study team towards the system being evaluated**		
